# Non-Targeted Dried Blood Spot-Based Metabolomics Analysis Showed Rice Bran Supplementation Effects Multiple Metabolic Pathways during Infant Weaning and Growth in Mali

**DOI:** 10.3390/nu14030609

**Published:** 2022-01-30

**Authors:** Brigitte A. Pfluger, Hillary V. Smith, Annika M. Weber, Hend Ibrahim, Lassina Doumbia, Abdoulaye Bore, Alima Cissoko, Seydou Douyon, Karim Kone, Lansana Sangare, Ababacar Maiga, Ousmane A. Koita, Kelli Goodman, Anne M. Evans, Elizabeth P. Ryan

**Affiliations:** 1 Nutrition and Health Sciences, Laney Graduate School, Emory University, Atlanta, GA 30322, USA; brigitte.pfluger@emory.edu; 2 Department of Global Health and Health Disparities, Colorado School of Public Health, Colorado State University, Fort Collins, CO 80523, USA; hillaryvsmith17@gmail.com; 3 Department of Food Science and Human Nutrition, Colorado State University, Fort Collins, CO 80523, USA; Annika.Weber@colostate.edu; 4 Department of Environmental and Radiological Health Sciences, Colorado State University, Fort Collins, CO 80523, USA; hendibrahim1@gmail.com; 5 Department of Medical Biochemistry, Faculty of Medicine, Zagazig University, Zagazig 44519, Egypt; 6 Laboratory of Applied Biology, Science and Technologies Faculty, University of Science, Techniques and Technologies of Bamako, Bamako E 3206, Mali; lassina.doumbia@lbma.edu.ml (L.D.); abdoubore@yahoo.fr (A.B.); Alimacissoko@yahoo.fr (A.C.); seydoudouyon522@yahoo.fr (S.D.); kkarbest@gmail.com (K.K.); lansana.sangare@lbma.edu.ml (L.S.); maigababacar@yahoo.fr (A.M.); okoita@icermali.org (O.A.K.); 7 Metabolon, Inc., Morrisville, NC 27560, USA; KGoodman@metabolon.com (K.G.); AEvans@metabolon.com (A.M.E.)

**Keywords:** rice bran, dried blood spots, metabolomics, nutrition, complementary feeding, Mali

## Abstract

Rice bran contains essential nutrients, antioxidants, and bioactives with anti-inflammatory and diarrheal protective properties important for infants. This 6-month randomized controlled trial investigated the effects of heat-stabilized rice bran supplementation during Malian infant weaning. Fifty healthy 6-month-old infants were randomized to a rice bran intervention (N = 25) or non-intervention control group (N = 25). Intervention infants received dose-escalating rice bran supplementation for 6 months (1–5 g/day). Monthly infant dried blood spot and anthropometric measurements were collected. Dried blood spot metabolite abundances were compared monthly according to diet for six months. Supplementation resulted in favorable weight-for-age and weight-for-length z-score changes. Non-targeted dried blood spot-based metabolomics identified 796 metabolites, of which 33% had significant fold differences between groups (7–12 months). Lipids and amino acids represented 70.6% of the metabolites identified. Rice bran supplementation during infant weaning significantly modulated the metabolites involved in antioxidant defenses and with neuroactive properties including reduced glutathione, glycine, glutamate, cysteinylglycine, tryptophan betaine, and choline. These findings support rice bran as a weaning ingredient to meet infant nutritional requirements and with the potential to reduce oxidative stress and improve cognitive outcomes. This study provides evidence for dried blood spots as a cost-effective tool to detect infant biomarkers of nutritional and metabolic status.

## 1. Introduction

The first 1000 days of life constitute a critical window when poor nutrition can negatively impact growth and development, cognition, and immune system function, among other factors. These are health outcomes that can be irreversible and have lasting impacts into adulthood [1,2,3,4]. Linear growth faltering, frequently referred to as stunting, is a sign of chronic malnutrition based on a child’s length for age. Wasting, on the other hand, indicates severe nutrient deficiency based on a child’s weight for length. Both result when a child is −2 standard deviations below the WHO Child Growth Standards median [5]. The greatest percentage of linear growth faltering occurs during the first 1000 days. Globally, of children under 5, roughly 41% of the estimated 149.2 million stunted children and 27% of the 45.4 million wasting children are in Africa [6].

Breastfeeding exclusively during the first 6 months of life, followed by complementary feeding with a variety of nutrient-dense foods from approximately 6 months to year two, can help ensure a child’s high nutritional demands are met [7,8,9]. Inadequate fulfilling of these needs is the case in many rural settings in low- and middle-income countries (LMICs) where complementary foods are in limited supply, unsafe (e.g., with high levels of mycotoxins), and not accessible nor affordable for timely introduction [10,11,12,13]. This is exemplified in Sub-Saharan Africa, where just 22% of children 6 to 23 months old meet the minimum dietary diversity requirements [14]. While many people advocate for animal source food consumption to meet an infant’s growing needs [7,15], these products (e.g., eggs, milk, meat) in LMICs are often expensive, have short shelf lives, or are unavailable in arid, poor, and food insecure areas and where livestock intensification has had adverse environmental impacts on water supply and soil fertility [10,16,17]. Furthermore, these animal-based foods do not help meet the total daily fiber intake requirements for establishing a healthy gut microbiome.

Grown in over 100 countries, rice is a leading cereal crop feeding over half of humanity [18]. Globally, 760 million tons of rice are produced annually, and approximately 81% of the 504 million tons of milled rice is used for human consumption [19]. Rice bran, or the outer layer of the rice grain, contains nutrients important for growth and development in the first 1000 days of life. It is rich in fiber, lipids, amino acids, and micronutrients such as thiamin (vitamin B1), niacin (vitamin B3), iron, and zinc, among others [20,21,22,23]. Many of the nutrients afforded by rice bran support gastrointestinal health, immunity, and brain development. Yet, the milling and processing of rice leaves the polished white rice grains depleted of these nutrients, and the bran is commonly used in animal feed or discarded [20,24]. Rice bran has demonstrated a number of health benefits in people across the lifespan, including as a nutrient-dense source of prebiotics and phytochemicals with antioxidant, anti-inflammatory, and chronic disease-fighting properties [25,26,27]. The potential benefits for the prevention of diarrheal disease via microbiome modulation further contribute to healthy guts and microbiomes [28,29,30,31]. As rice is a commonly consumed staple crop in food insecure countries across Africa, the potential for rice bran’s nutritional scalability and sustainability is of notable and remarkable importance.

Mali is a landlocked country in West Africa with food insecurity among approximately 25% of the population [18]. An estimated 26% of Malian children aged 0 to 4 years old are stunted, and only 22% of children aged 6 to 23 months consume a minimum diet diversity. With regards to breastfeeding in Mali, less than half of children under 6 months old are exclusively breastfed (40%), while an estimated 59% are introduced to complementary feeding from 6 to 8 months of age [14].

Non-targeted metabolomics analysis quantifies all detectable metabolites across a large suite of biological matrices that may include, but not be limited to, tissues, cells, blood compartments, urine, and feces. Dried blood spots (DBS) is a biological sampling technique in which a few blood droplets collected via heel or fingerpricks are applied to filter paper and air dried [32]. DBS is relatively non-invasive, easy-to-use, and less expensive when compared to other blood sample collection methods, thus providing potential for widespread assessment of infant metabolic profiles in community and field settings.

This study investigated the effects of dietary heat-stabilized rice bran supplementation in Mali during healthy infant weaning and utilized DBS to identify novel nutrition and metabolic biomarkers via non-targeted metabolite profiling from 6 to 12 months of age. As a secondary aim, this trial identified changes in infant growth and hemoglobin measures as a result of daily dietary rice bran supplementation.

## 2. Materials and Methods

### 2.1. Study Population

**Figure 1:**
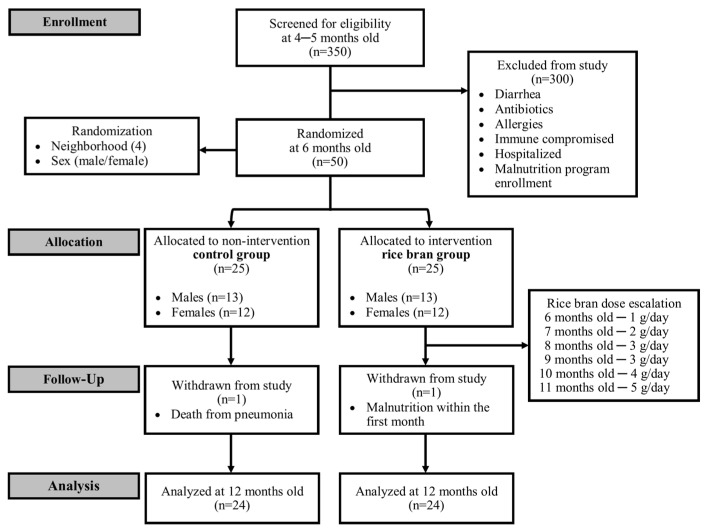
Study recruitment and participation. The 6-month-old infants (*N* = 50) recruited from Dioro, Mali were randomized by sex and location (neighborhood) to one of two study arms: non-intervention control group or rice bran intervention group.

This 6-month randomized controlled dietary intervention trial conducted in Dioro, Mali, assessed the feasibility of incorporating increasing amounts of heat-stabilized rice bran (Calrose-M202 USA variety) into the infant diet from ages 6 to 12 months. In total, 350 infants between 4 and 5 months of age were assessed for eligibility at the Dioro Community Health Center prior to enrollment to ensure they were healthy (Figure 1). Of those screened, 50 healthy infants were recruited and randomized based on location (i.e., neighborhood) and sex to the rice bran intervention group (N = 25) or the non-placebo, non-intervention control group (N = 25). Infants were excluded from the study if they had a diarrhea episode and/or used antibiotics in the month prior to study onset, had known allergies, were immune compromised, had been hospitalized, or had been enrolled in a malnutrition treatment program [33]. Table 1 shows participant demographics for infants enrolled in both groups at baseline (i.e., 6 months of age).

### 2.2. Intervention Dosing and DBS Collection Procedures

Infant DBS and anthropometric measurements were collected monthly using standard procedures, as previously described [33,34]. Those in the intervention group received daily rice bran supplementation, which increased incrementally throughout the study period starting with 1 g of rice bran at baseline (6 months of age). Supplementation then increased to 2 g daily at 7 months of age, 3 g daily at 8 and 9 months, 4 g daily at 10 months, and then 5 g at 11 months of age (Figure 1). Rice bran dosing was set well below the recommended limit to ensure safety and tolerability. Dietary intake data for weaning foods consumed were collected prospectively as self-reports from the parents/guardians, but intake was not quantified. Compliance of rice bran-fed infants was high throughout the study (99%), and health-care workers visited households daily to ensure compliance, monitor health status and diarrheal incidence, and assess antibiotic use [33]. Rice bran was consumed by infants either directly or in combination with complementary foods. This included mothers feeding infants rice bran mixed with water (46%), directly (18%), or with grain porridge (25%), such as millet, sorghum, or rice. Water quality and safety checks were conducted, as water-borne infections can further contribute to diarrhea and nutrient loss [35]. DBS were collected from children on a monthly basis and stored at 25–30 °C (77–86 °F) and 55% relative humidity before being shipped to Colorado State University (Supplementary Table S1). Finger pricks with BD Ultra-fine II 30-gauge lancets were completed by study coordinators at each visit. DBS were collected on filter paper (GE Health Care Whatman Card) and sent to Metabolon, Inc. (Morrisville, NC, USA), for elution, extraction, and metabolomics. Hemoglobin was collected separately from the DBS using the HemoCue^®^ Hb301.

### 2.3. DBS Metabolic Profiling

DBS samples were received and inventoried by Metabolon and stored at −20 °C until processing. Two 6 mm punches were taken from each DBS sample and extracted together in a 2 mL 96-well plate. First, to reconstitute the dried blood and facilitate the recovery of metabolites from the filter paper, a small aliquot of water was added to DBS punches, followed by two minutes of vigorous shaking on a Glen Mills GenoGrinder 2000 and a brief centrifugation step. The proteins were then precipitated with methanol, which had been spiked with recovery standards for quality control purposes, and then samples were placed on the GenoGrinder again for four minutes before another centrifugation step to separate the protein and filter paper particulates from the solution containing the extracted metabolites. The supernatant was divided into five portions using a Hamilton MicroLab STAR^®^ automated liquid handling system and dried completely under a steady stream of nitrogen gas using a TurboVap^®^ (Zymark). One portion was reserved for backup purposes, while the other four were reconstituted with the appropriate solvent for the chromatographic method on which it was ran. The four analytical methods are as follows: two separate reversed-phase ultra-high-performance liquid chromatography–tandem mass spectrometry (RP/UPLC–MS/MS) methods in positive electrospray ionization mode (ESI+), one RP/UPLC–MS/MS method in negative electrospray ionization mode (ESI−), and one hydrophilic interaction chromatography (HILIC)/UPLC–MS/MS method in ESI-. All four methods used a Waters ACQUITY UPLC and a Thermo Scientific Q-Exactive high-resolution mass spectrometer with a heated electrospray ionization source and Orbitrap mass analyzer set at 35,000 mass resolution. Detailed liquid chromatography (LC) and MS parameters can be found in the work of Ford et al. (2020) [36].

Technical replicates of a DBS QC sample that was prepared at Metabolon by spotting a large batch of cards with the same lot of whole blood was extracted in each plate and injected periodically throughout the platform run to monitor the overall process variability of endogenous biochemicals. Variability of the internal standards that were spiked in the reconstitution solvents and added to each sample immediately before analysis was monitored across all experimental samples to assess platform variability. A number of curation steps and quality control processes were taken to ensure that the resulting data used for statistical analysis and interpretation was accurate and consistent in identifying chemical compounds, and to ensure that compounds representing system artifacts, miss-assignment, and background noise were removed. Proprietary visualization and interpretation software were used to check for consistency of peak identification in the samples, and library matches for each compound were checked for each sample as well as adjusted when needed.

Four major informatics system components, founded on Local Area Network (LAN) backbone and a database server running Oracle 10.2.0.1 Enterprise Edition, were utilized by analysts in this study: LIMS, data extraction and peak-identification software, data processing tools for quality control and compound identification, and various information interpretation and visualization tools.

### 2.4. Statistical Analysis

After batch normalization, missing values were imputed with the minimum value on a per metabolite basis, and then (natural)-log transformed prior to running a two-way repeated measures ANOVA. Pairwise t-tests of the least squared mean differences were performed for rice bran and control infants at months 7 to 12 compared to baseline (month 6) and comparing rice bran-fed to control subjects at each time point. Pairwise changes were summarized for all statistical comparisons using fold changes/differences of the exponentiated least squares means. Metabolites with a statistical difference between rice bran-fed and control infants at baseline only were removed from analysis. A fold difference identifies metabolites between control and rice bran groups at a single timepoint, and a fold change term represents a metabolite change over time during infant growth. Statistical metabolite analysis was performed using Omicsoft Array Studio version 7.2, and fold changes/differences were calculated using Metabolon’s LIMS software. Anthropometric and hemoglobin measures were analyzed via difference-in-differences analysis to compare group outcomes at two study timepoints and using SAS/STAT^®^ software version 9.4. The clustered heat map was designed in RStudio version 4.1.0. *P*-value determinations for statistical significance were set at *p* ≤ 0.05 for all tests. When excluding all insufficient volume DBS samples, fewer statistically significant results were observed for the comparisons between the rice bran and control groups across all time points. Thus, insufficient DBS volume samples were excluded from analysis. To account for multiple comparisons, false discovery rates were estimated using Storey’s q-value method, as previously reported [37]. A false discovery rate of q < 0.10 indicated high confidence in a result after p-value determinations for significance. Data visualization of median-scaled relative abundance (MSRA) changes and ANOVA comparisons of study groups over time were created using GraphPad Prism version 8.3.1.

## 3. Results

### 3.1. Anthropometric and Hemoglobin Measurements

This trial investigated the effects of heat-stabilized rice bran supplementation on length-for-age (LAZ), weight-for-age (WAZ), and weight-for-length (WLZ) z-scores in weaning infants from 6 to 12 months of age, controlling for sex and neighborhood (Table 2; Supplementary Figure S1). No significant LAZ differences were seen from baseline among rice bran-fed infants at age 12 months compared to control. Significant differences in WAZ were seen in rice bran-fed infants relative to the control compared to baseline from months 8 (0.42 difference, *p* = 0.029) through 12 (0.66 difference, *p* = 0.003) and in WLZ from months 10 (0.61 difference, *p* = 0.023) through 12 (0.67 difference, *p* = 0.008). Statistical significance for WAZ and WLZ strengthened as the trial progressed. Significant differences were also seen in rice bran-fed infants relative to the control at month 10 for hemoglobin (g/dL) (0.86 difference, *p* = 0.031), when compared to baseline.

### 3.2. Principal Component Analysis

**Figure 2:**
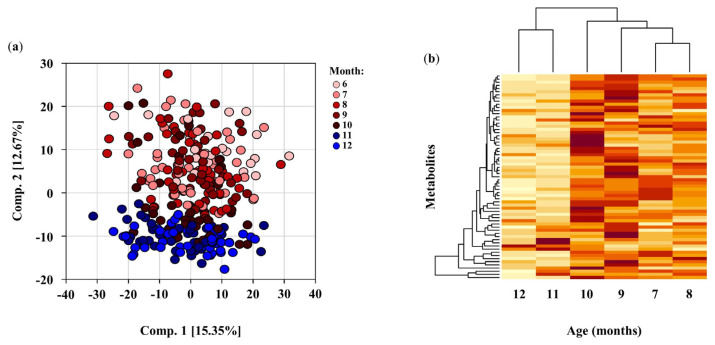
(**a**) Principal component analysis (PCA) of all infant participant DBS metabolites at each month of age (6–12). Clear separation of component 2 (comp.2) between early stage of weaning (6–9 months of age) versus later stage of weaning (11–12 months of age). Month = infant month of age. Comp. = component. (**b**) Hierarchal clustering visualized as a heat map for all metabolites with significantly higher or lower fold differences (*p* ≤ 0.05) comparing rice bran-fed to control infants during at least one timepoint from months 7–12. A fold difference identifies metabolites between control and rice bran groups at a single timepoint. Darker color bars represent metabolites with higher fold differences. Significant fold differences show a cluster separation at 9 and 10 months of age. P-values were calculated by two-way repeated measure ANOVA comparisons.

The principal component analysis (PCA) of the DBS metabolome in all children resulted in a clear separation in component 2 between weaning in early stages from 6–9 months of age and later weaning stages (11–12 months of age) (Figure 2a). Three outliers were removed from this analysis. There was strong evidence for separation by infant age in the metabolic profiles of all weaning infants enrolled in this study. There were no observable gender-based separations.

### 3.3. Infant DBS Metabolomics from 6–12 Months of Age

In total, 796 metabolites were identified in infant DBS across eight chemical classes: lipids, amino acids, xenobiotics, nucleotides, carbohydrates, peptides, vitamins and cofactors, and energy metabolites (Supplementary Table S2). We identified 103 (12.94%) significant differences in the relative abundance of DBS metabolites between groups at baseline. Excluding these differences at baseline, Figure 2b includes 229 (33%) metabolites that were significant for having fold differences when comparing rice bran-fed to control infants at 7 to 12 months of age. Cluster analysis by the heat map revealed that a number of those significant metabolite differences occurred at 9 and 10 months of age (Figure 2b). Non-targeted metabolomics identified 65 metabolites with both significantly higher fold changes and fold differences during at least one timepoint. The largest percentages of metabolites with significant fold changes and fold differences at the same timepoints were observed at 8, 9, and 10 months of age.

**Figure 3:**
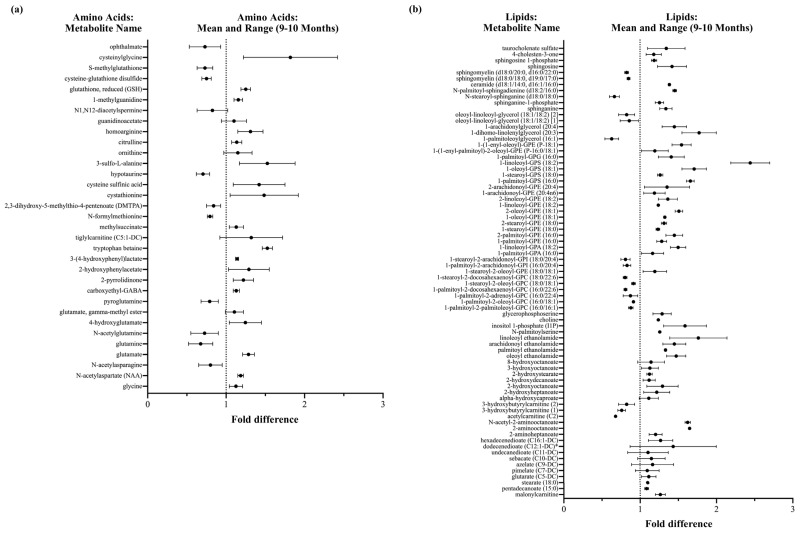
Major shifts were observed in DBS amino acids and lipids following rice bran supplementation in weaning infants at 9–10 months of age. (**a**) Amino acid metabolites with significant fold differences (increase or decrease) for rice bran-fed compared to control infants; (**b**) Lipid metabolites with significant fold differences (increase or decrease) for rice bran-fed compared to control infants. The mean and range are shown. Metabolites are listed in order by sub-pathway (see Supplementary Table S2). A fold difference revealed metabolites between control and rice bran groups at a single timepoint.

Infant DBS metabolomics showed that rice bran supplementation during infant weaning impacted antioxidant defenses, vitamin and co-factor pathways, lipid profiles, phytochemical and microbial systems, and neuroactive pathways. Collectively, amino acids and lipids represented the largest proportion (70.6%) of DBS metabolites identified. A large segment of the amino acid and lipid metabolites that significantly changed (increased or decreased) at 9 and 10 months of age had higher fold differences for rice bran-fed compared to control infants (Figure 3). 

#### 3.3.1. DBS Molecules Involved in Antioxidant Defenses

**Figure 4:**
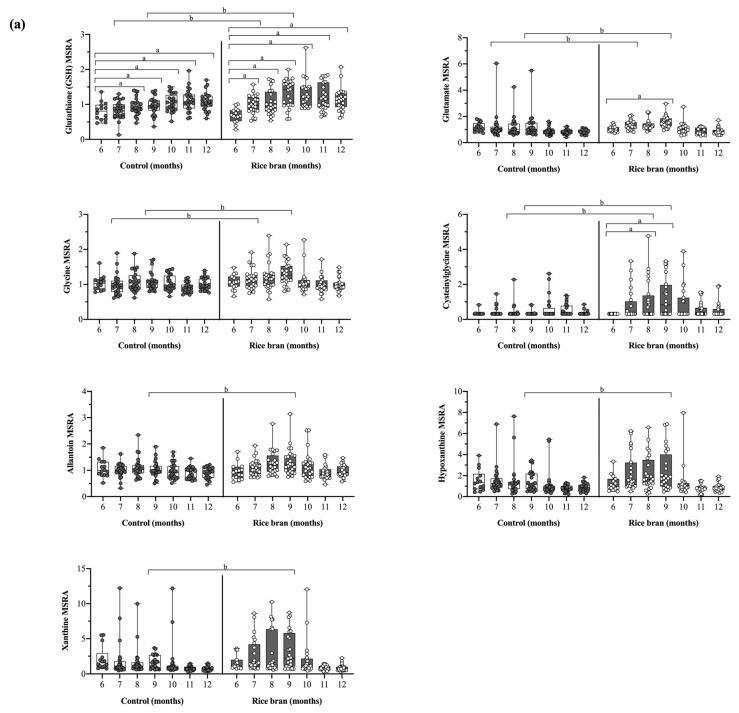

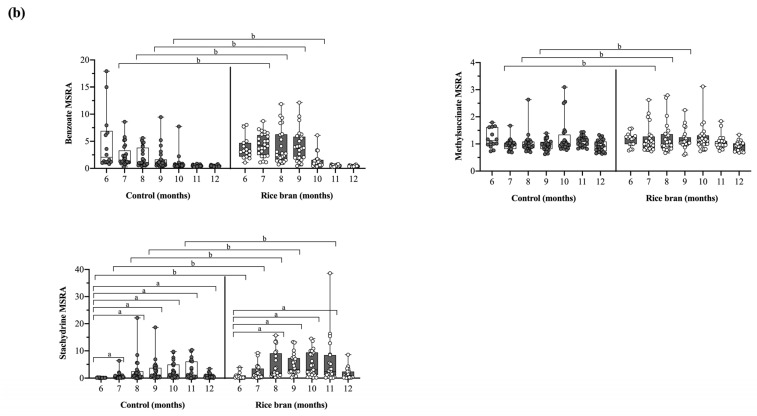
Box plots of the median-scaled relative abundance (MSRA) for each metabolite over time for rice bran-fed and control infants by infant age (6 to 12 months old). DBS metabolites representing (**a**) antioxidant defenses and (**b**) phytochemical and microbial metabolites are shown. Significant differences are highlighted at each timepoint when compared to baseline within groups (fold change) and at one time point for comparison between groups (fold difference). a = significantly higher fold change within a group (*p* ≤ 0.05). b = significantly higher fold difference for rice bran compared to control infants (*p* ≤ 0.05). *P*-values were calculated by two-way repeated measure ANOVA comparisons.

There is a collection of small molecules involved in antioxidant defenses that are important for infant growth, immunity, and protection against harmful exposures. Antioxidants work to reduce free radicals and stop oxidative stress, and an imbalance in such systems can lead to DNA, protein, and lipid damage as well as neurological and chronic inflammatory diseases, among others. The modulation of antioxidant defenses was reflected in alterations to the glutathione oxidation and reduction pathways following supplementation. As shown in Figure 4a, compared to the control, rice bran-fed infants had similar or slightly lower MSRA at baseline of reduced glutathione (GSH) and its precursors glutamate, glycine, and cysteinylglycine. These metabolites increased from 6 to 9 months of age. Significant fold differences between groups, and with higher levels for rice bran-fed compared to control infants, were seen at months 7 and 9 for reduced glutathione (1.31-fold, *p* = 0.0031), glutamate (1.36-fold, *p* = 0.0013), and glycine (1.21-fold, *p* = 0.0042), and at months 8 and 9 for cysteinylglycine (2.42-fold, *p* < 0.0001). Compared to baseline, the fold change for reduced glutathione significantly increased in rice bran-fed infants at all timepoints and in control infants at 8 through 12 months of age. The fold change for glutamate also increased significantly but only for rice bran-fed infants at month 9 (1.29-fold, *p* = 0.0231).

Hypoxanthine and xanthine are precursors of uric acid, a powerful antioxidant, and allantoin is a product of uric acid oxidation and an oxidative stress biomarker [38]. Compared to control infants, hypoxanthine and xanthine MSRA were higher in rice bran-fed infants from months 7 to 10. Furthermore, at 9 months of age, the fold difference for hypoxanthine (1.46-fold, *p* = 0.0391), xanthine (1.56-fold, *p* = 0.0276), and allantoin (1.23-fold, *p* = 0.0213) were significantly higher in rice bran-fed compared to control infants (Figure 4a).

#### 3.3.2. DBS Phytochemicals and Gut Microbial Derived Molecules

DBS showed significant differences between groups in microbial- and phytochemical-derived metabolites (Figure 4b). The fold difference for benzoate, which can reflect the microbiota-assisted breakdown of plant-derived phenolic compounds, was significantly higher at months 7 (2.12-fold, *p* = 0.0001), 8 (2.08-fold, *p* = 0.0002), 9 (2.71-fold, *p* < 0.0001), and 10 (1.54-fold, *p* = 0.0281) in rice bran-fed compared to control infants. Methylsuccinate, a metabolite with possible metabolic connections to the microbiome, had a significantly higher fold difference in rice bran-fed compared to control infants at months 7 (1.18-fold, *p* = 0.0377), 8 (1.18-fold, *p* = 0.0454), and 9 (1.22-fold, *p* = 0.0158). The fold difference for the phytochemical stachydrine was significantly higher in rice bran-fed compared to control infants at months 7 (2.60-fold, *p* = 0.0138), 8 (2.54-fold, *p* = 0.0175), 9 (2.46-fold, *p* = 0.0216), and 11 (2.52-fold, *p* = 0.0205).

#### 3.3.3. DBS Vitamin and Co-Factor Molecules

**Figure 5:**
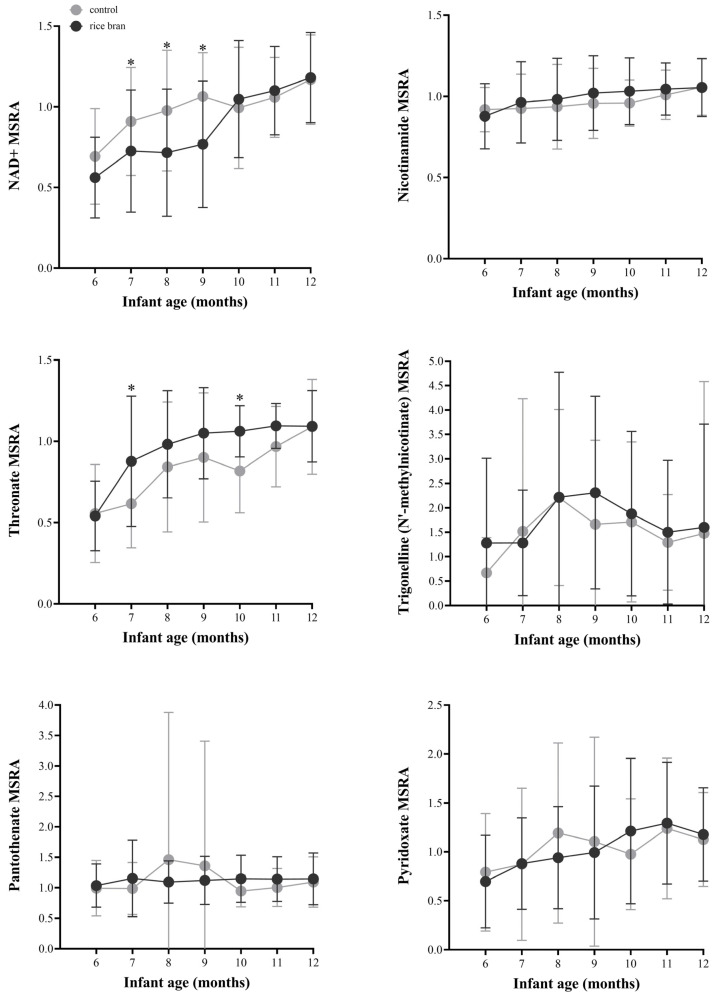
Line plots of the median-scaled relative abundance (MSRA) for each metabolite over time for rice bran-fed and control infants by infant age (6 to 12 months old). DBS metabolites representing vitamin and cofactors are shown. Significant fold changes between rice bran-fed and control infants are depicted at each timepoint. * = significant fold difference for rice bran compared to control infants (*p* ≤ 0.05). *P*-values were calculated by two-way repeated measure ANOVA comparisons.

In total, there were 14 vitamin and cofactor metabolites across five sub-pathways identified in DBS. Significant changes were identified for metabolites involved in nicotinate and nicotinamide metabolism (Figure 5). Compared to control, rice bran-fed infants showed slightly elevated MSRA for the coenzyme nicotinamide adenine dinucleotide (NAD+) from 10 to 12 months of age, despite lower MSRA in earlier months. Compared to baseline, significant fold changes in NAD+ were seen for rice bran-fed infants at months 10 (2.05-fold, *p* = 0.0001), 11 (2.38-fold, *p* < 0.0001), and 12 (2.43-fold, *p* < 0.0001) and for control infants at months 9 (1.54-fold, *p =* 0.0082), 11 (1.71-fold, *p =* 0.0012), and 12 (1.55-fold, *p =* 0.0080) (Supplementary Table S2). Nicotinamide is a functional group component of NAD+ that showed a slightly higher MSRA for rice bran-fed infants from 7 to 11 months of age, but without reaching statistical significance.

Compared to baseline, significant fold change increases in the cofactor threonate were seen in rice bran-fed infants at months 7 through 12 (2.13-fold, *p* < 0.0001) and control infants at months 8 through 12 (1.92-fold, *p =* 0.002). Rice bran-fed compared to control infants also had significantly higher fold differences in threonate at months 7 (1.41-fold, *p =* 0.0070) and 10 (1.31-fold, *p =* 0.0398). Trigonelline (N’-methylnicotinate), a product of niacin metabolism, showed a higher MSRA in rice bran-fed compared to control infants from 9 to 12 months of age, despite a lower MSRA at 7 months of age.

Variations were observed between rice bran-fed and control infants in the MSRA for the vitamins pantothenate (B5) and pyridoxate (B6) (Figure 5). The MSRA for pantothenate in rice bran-fed infants was similar throughout the study months, whereas control infants showed fluctuations. For pyridoxine, the MSRA in rice bran-fed infants steadily increased from 6 to 11 months and decreased in the last month while levels in control infants fluctuated over time. When compared to baseline, significant fold changes for pyridoxate were observed in control infants at months 8 (1.65-fold, *p =* 0.0293), 11 (1.76-fold, *p =* 0.0151), and 12 (1.76-fold, *p =* 0.0112).

#### 3.3.4. DBS Lipid Components and Metabolic Pathways

There were 382 lipid metabolites across 47 sub-pathways identified, accounting for the largest percentage (48%) of all infant DBS metabolites. Of these, 39 can be found in rice bran (Supplementary Table S2) [22].

**Figure 6:**
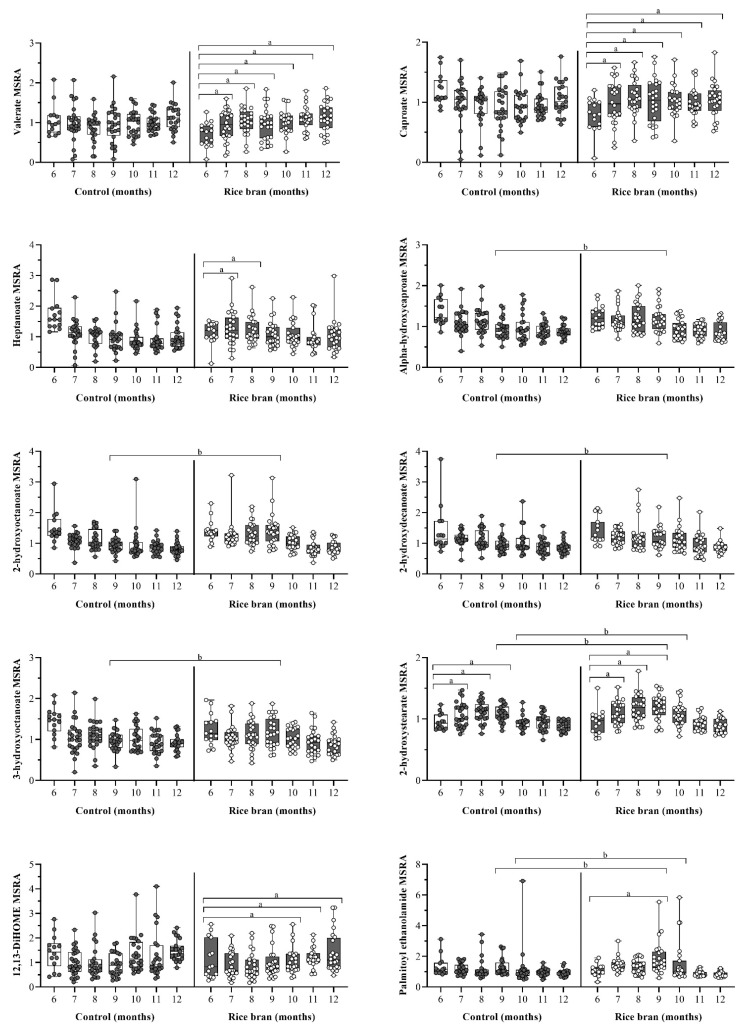
Box plots of the median-scaled relative abundance (MSRA) for each metabolite over time for rice bran-fed and control infants by infant age (6 to 12 months old). DBS metabolites representing lipids are shown. Significant differences are highlighted at each timepoint compared to baseline within groups (fold change) and at one time point for comparison between groups (fold difference). **a** = significantly higher fold change within a group (*p* ≤ 0.05). **b** = significantly higher fold difference for rice bran compared to control infants (*p* ≤ 0.05). *P*-values were calculated by two-way repeated measure ANOVA comparisons.

DBS-based non-targeted metabolomics revealed changes to short- and medium-chain fatty acids. Compared to baseline, the fold change for the short-chain fatty acid valerate was significantly higher in rice bran-fed infants at all timepoints, while no significant changes were seen in control infants during the study period. Among the medium-chain fatty acids, rice bran-fed compared to control infants showed gradual increases in MSRA for caproate and heptanoate. Within group comparisons to baseline showed the fold change for caproate significantly increased in rice bran-fed infants at all timepoints, whereas it significantly decreased in control infants at months 7 (0.73-fold, *p =* 0.0285), 8 (0.73-fold, *p =* 0.0306), and 9 (0.70-fold, *p =* 0.0121). Compared to baseline, the fold change for heptanoate significantly increased in rice bran-fed infants at months 7 (1.48-fold, *p =* 0.0108) and 8 (1.49-fold, *p =* 0.0093) and significantly decreased in control infants at all timepoints (Figure 6).

Five monohydroxy fatty acids (Figure 6) potentially derived from rice bran were identified in infant DBS: alpha-hydroxycaproate, 2-hydroxyoctanoate, 2-hydroxydecanoate, 2-hydroxystearate, and 3-hydroxyoctanoate. Significant fold differences between groups were seen at month 9 for alpha-hydroxycaproate (1.24-fold, *p =* 0.0034), 2-hydroxyoctanoate (1.50-fold, *p* < 0.0001), 2-hydroxydecanoate (1.20-fold, *p =* 0.0280), and 3-hydroxyoctanoate (1.24-fold, *p =* 0.0208), and for 2-hydroxystearate at months 9 (1.09-fold, *p =* 0.0481) and 10 (1.16-fold, *p =* 0.0017). Compared to baseline, DBS showed significant fold change increases for 2-hydroxystearate at months 7 through 9 for rice bran-fed (1.23-fold, *p =* 0.0006) and control (1.14-fold, *p =* 0.0165) infants.

DBS revealed fold change shifts in other noteworthy rice bran-associated lipid metabolites. Compared to baseline, the dihydroxy fatty acid 12, 13-DiHOME significantly increased in rice bran-fed infants at months 10 through 12 (1.93-fold, *p =* 0.0007) and significantly decreased in control infants at month 9 (0.70-fold, *p =* 0.0335). Compared to control, rice bran-fed infants had significantly higher levels of palmitoyl ethanolamide (PEA) at months 9 (1.35-fold, *p =* 0.0077) and 10 (1.32-fold, *p =* 0.0147). Within group comparisons to baseline showed that the fold change for PEA was significantly lower in control infants at months 10 through 12 (0.66-fold, *p =* 0.0120) and was significantly higher in rice bran-fed infants at month 9 (1.34-fold, *p =* 0.0449) (Figure 6).

Rice bran-fed infants also showed increases in eicosapentaenoate (EPA), docosahexaenoate (DHA), arachidonate, linoleate, and linolenate at month 9. For most of these lipids, the rice bran group started at slightly lower baseline levels compared to the control, but without statistical differences identified between study groups (Supplementary Table S2).

#### 3.3.5. Rice Bran Supplementation and Neuroactive Molecules

**Figure 7:**
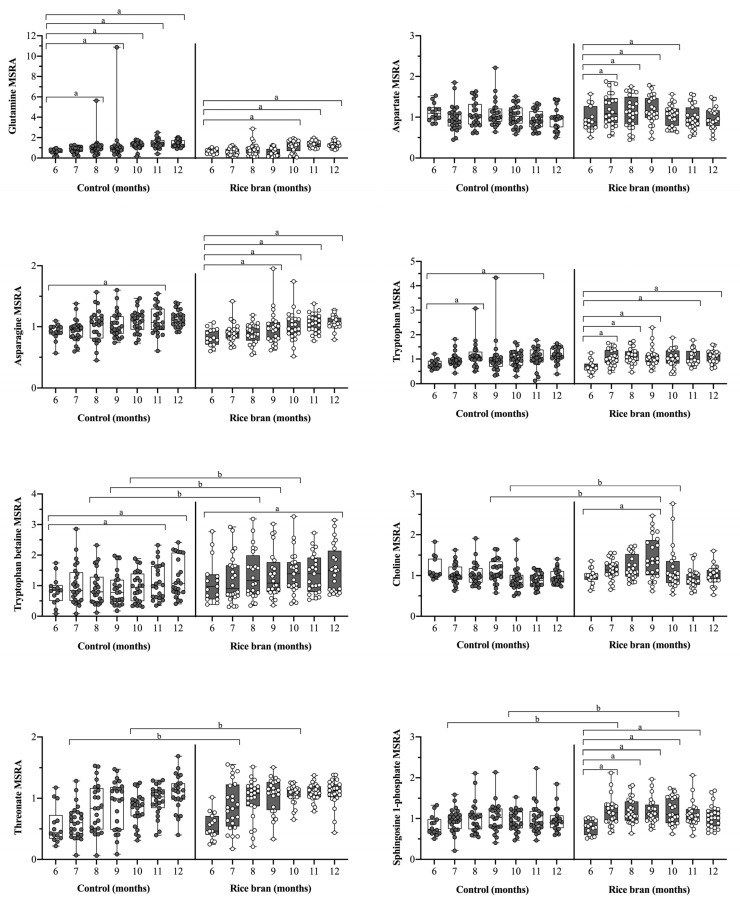
Box plots of the median-scaled relative abundance (MSRA) for each metabolite over time for rice bran-fed and control infants by infant age (6 to 12 months old). DBS metabolites representing neuroactive properties are shown. Significant differences are highlighted at each timepoint compared to baseline within groups (fold change) and at one time point for comparison between groups (fold difference). **a** = significantly higher fold change within a group (*p* ≤ 0.05). **b** = significantly higher fold difference for rice bran compared to control infants (*p* ≤ 0.05). *P*-values were calculated by two-way repeated measure ANOVA comparisons.

Nutrition is key for optimal brain and cognitive development. DBS-based metabolites with neuroactive implications include the amino acids glutamate and glycine, the results of which have already been discussed, as well as glutamine, aspartate, and asparagine, all of which are found in abundance in the central nervous system (Figure 7) [39,40,41,42]. Compared to baseline, the fold change for glutamine significantly increased in rice bran-fed infants at months 10 through 12 (2.29-fold, *p* < 0.0001) and in control infants at months 8 through 12 (2.21-fold, *p* < 0.0001). Aspartate increased significantly among rice bran-fed infants, compared to baseline, at months 7 through 10 (1.21, *p =* 0.0157), while no significant changes were seen in control infant DBS. Compared to baseline, asparagine increased significantly among control infants at month 11 (1.17-fold, *p =* 0.0108), yet rice bran-fed infants showed significant increases in this metabolite at months 9 through 12 (1.32-fold, *p =* 0.0001).

Other DBS neuroactive metabolites with noteworthy outcomes are depicted in Figure 7. Compared to baseline, tryptophan, an amino acid needed to endogenously synthesize niacin and the neurotransmitters serotonin and melatonin [43], increased significantly for control infants at months 8 (1.35-fold, *p =* 0.0113) and 11 (1.39-fold, *p =* 0.0051) and for rice bran-fed infants at all timepoints excluding 10 months of age. Tryptophan betaine, which is involved in tryptophan metabolism, showed significant between group fold differences at months 8 through 10 (1.46-fold, *p =* 0.0325), and with rice bran-fed infants having higher levels.

Choline is another nutrient present in rice bran that aids in neurotransmission, and deficiency can impair cognitive function [44,45]. Compared to baseline, the fold change for choline significantly increased for rice bran-fed infants at month 9 (1.28-fold, *p =* 0.0032) and significantly decreased in control infants at months 10 through 12 (0.78-fold, *p =* 0.0001). The fold difference for choline was significantly higher for rice bran-fed infants compared to control at months 9 (1.25-fold, *p =* 0.0043) and 10 (1.23-fold, *p =* 0.0093). Between group fold differences were also observed for the rice bran-associated metabolites threonate at months 7 (1.41-fold, *p =* 0.0070) and 10 (1.31-fold, *p =* 0.0398), and for sphingosine 1-phosphate at months 7 (1.23-fold, *p =* 0.0162) and 10 (1.22-fold, *p =* 0.0233).

## 4. Discussion

It is well understood that adequate nutrition plays a significant role during infant development, including for optimal brain and cognitive functions during the first 1000 days of life [46]. Heat-stabilized rice bran represents a promising, affordable, and novel food ingredient for complementary feeding during weaning and to sustainably improve household nutritional status, ultimately contributing to better growth outcomes and malnutrition reductions in LMICs. The DBS-based non-targeted metabolomics used in this study for measuring metabolic responses to rice bran supplementation clearly highlighted the roles of lipids and amino acids, which collectively represented 70.6% of the metabolites identified in samples. These nutrients play critical roles in countries such as Mali, where starch-based diets include processed grains such as rice and millet, yet are inadequate in key lipids, amino acids, micronutrients, vitamins, and minerals offered by rice bran. The prebiotics and fiber profile of rice bran alongside its phytochemicals are especially important sources of substrates to the developing infant gut microbiota. Infant gut microbiota are often altered by pathogens and less mature in composition for vulnerable populations at risk for malnutrition.

Several metabolites identified from infant DBS, such as benzoate, valerate, caproate, and palmitoyl ethanolamide, have linkages to metabolism by gut microbiota. DBS metabolites may also represent components found in the rice bran food and that carry important gut health properties. Examples include the amino acids tryptophan, glutamine, and glycine, which can positively impact the gut microbiome, including its composition and influencing intestinal barrier function [47,48,49]. These DBS metabolites, along with others identified in the rice bran food provided to infants during weaning, merit exploration alongside gut microbiota shifts during growth.

The food metabolome of the Calrose rice bran variety used in this feeding trial was previously reported to contain 421 identified metabolites that include lipids, amino acids, carbohydrates/energy, nucleotides, cofactors and vitamins, secondary metabolites, and peptides [22]. Overlapping metabolites from the Calrose rice bran food with the infant DBS included 26 lipids, 55 amino acids, 18 carbohydrates/energy, and 10 peptides. Of these 109 potentially rice bran-derived metabolites identified in the DBS, 93 metabolites were significant according to diet and timepoint interaction analysis. This finding emphasizes bioavailability of rice bran components via the direct co-analysis of the food molecules that may be present in the infant DBS metabolome.

This feeding trial demonstrated evident changes to lipid, antioxidant, and neuroactive pathways. Daily rice bran supplementation shifted infant metabolism at 9 months of age, which is a critical window in child development. The 9-month-old developmental timepoint is key for dietary exposures as infants advance in gross and fine motor skills such as standing and grasping objects, among others [50]. The age-dependent shifts for reduced glutathione (GSH), glutamate, and cysteinylglycine for rice bran-fed infants and its precursors, as well as other key antioxidants, also suggest that rice bran-fed infants may experience lower levels of oxidative stress. These findings have implications for reducing the risk of major chronic diseases, such as cardiovascular disease and neurodegeneration later in life that have been linked to oxidative stress [51,52]. Additionally, the DBS metabolites 2-hydroxystearate, palmitoyl ethanolamide, tryptophan betaine, choline, and sphingosine 1-phosphate all showed significantly higher fold changes and differences for rice bran-fed infants. The promising potential for rice bran to offer neuroprotection through neuroactive metabolites will be critical for future studies designed to make linkages between nutrition, the gut microbiome, and neurodevelopment, including cognition. Metabolites from this investigation merit validation in other country cohorts in order to serve as infant biomarkers of nutritional and metabolic status according to age.

Linear growth faltering is a consequence of long-term undernutrition. The physical benefits of dietary interventions on weight-related measures are generally apparent in shorter time periods than those of linear-related measures, while linear growth faltering improvements are typically evident in longer-term (e.g., at least a year) trials. Shorter-term studies frequently have no anthropometry or include unclear LAZ results in this regard [53,54,55]. The growth improvements detected in WAZ and WLZ from this study could be co-attributed to the lower diarrheal incidence, which was 33% for rice bran-fed infants compared to 79% for control infants [33]. Higher incidence of diarrheal episodes in the control group could have resulted in higher nutrient loss and lower nutrient absorption that results in a slower rate of weight gain, all of which are involved in growth faltering in this region. Rice bran’s protective effects against diarrhea and its potential to boost gut mucosal immune function could alleviate enteric infections and boost the delivery, absorption, and utilization of key nutrients and metabolites critical for infant growth, vaccine efficacy, and cognition, among others.

This study had limitations including the small number of participants and the lack of infant weaning food logs that did not measure or quantify other common sources of local weaning foods consumed. Dietary intake was collected prospectively by parents/guardians via self-reported food logs. No differences were found in the types of foods provided to all participants during the infant weaning period. The contribution of other complementary weaning foods with rice bran supplementation does merit attention in follow-up studies with respect to the WAZ and WLZ changes observed over time. Dietary biomarkers of food exposure and intake diaries for weaning infants are challenging to quantify yet should be applied in future studies to make associations with observed anthropometric changes. Additional studies are also warranted to address mechanisms of action related to enhanced nutrient absorption with rice bran [56]. For example, the metabolite concentrations of fat-soluble vitamins (A, D, E, and K) may be improved following ingestion of rice bran lipids that may aid in absorption. Although DBS are recognized as comprehensive blood samples in global health research [32], some DBS limitations include sample collection variability, such as blood spot volume during collection, and inconsistent storage conditions, including transportation [32,57]. In remote or resource-poor settings, DBS sample collection and metabolomics may be challenging due to a lack of access to supplies (e.g., DBS cards), machinery (e.g., mass spectrometer), and capacity to process and run the samples. The generalizability of DBS metabolomics results may be another limitation with respect to reproducibility across countries for these reasons [58].

## 5. Conclusions

Our data analysis of DBS-based non-targeted metabolomics points to the potential for heat-stabilized rice bran supplementation to positively improve weaning infant nutritional outcomes important in the first 1000 days of life. Rice bran impacted multiple metabolic pathways, including infant antioxidant defenses, lipid profiles, and neuroactive molecules. DBS-based metabolomics were sensitive to support rice bran as a food ingredient providing important nutrients during weaning, which is especially influential in LMIC settings where multi-nutrient deficiency and food insecurity are common. Supplementation also positively impacted child growth measures, as seen in the favorable changes in WAZ and WLZ among rice bran-fed infants.

Rice bran is an underutilized crop co-product from processing/milling that could provide integrated agricultural/nutritional diversification while reducing food waste. Furthermore, heat-stabilized rice bran product development for human consumption requires limited additional energy or land use, thus contributing to environmental sustainability. The interplay of rice bran occurs with roles in environmental, economic, and nutritional contexts, and this powerful, nutrient-dense food may improve global food security through valorization. Coordinated regional and multi-national agricultural and health strategies are necessary for sustainable production and adoption of rice bran based on the local context.

## Figures and Tables

**Table 1:** Participant demographics for both study groups. Data shown are from control and rice bran-fed Malian infants at baseline (6 months of age).

	Control (*N* = 24)	Rice Bran (*N* = 24)
**Sex (%)**		
Female	12(50)	12(50)
Male	12(50)	12(50)
**Mother’s education (%)**		
None	12(50)	11(46)
Some primary	4(17)	7(29)
Completed primary	6(25)	1(4)
Some secondary	1(4)	2(8)
Completed secondary	1(4)	3(13)
University	0(0)	0(0)
**Breastfeeding status (%)**		
6 months	24(100)	24(100)
**Sanitation systems (%)**		
Community latrine	21(87.5)	19(76)
Private latrine	3(12.5)	5(20.8)
**House type (%)**		
Mud	16(66)	17(70)
Sheet metal	5(20)	5(21)
Cement	3(14)	2(8)
**Water source (%)**		
Untreated ground water	24(100)	24(100)
**Water risk assessment (%)**		
Safe	7(44)	3(13)
Low risk	2(9)	2(8)
Low-mid risk	4(17)	5(21)
Mid-high risk	2(9)	2(8)
High risk	4(17)	6(25)
Unsafe	5(21)	6(25)
**Anthropometry (mean ± SD)**		
Weight (kg)	7.02 ± 0.88	7.14 ± 0.99
Length (cm)	65.57 ± 2.56	66.56 ± 3.12
Weight-for-age Z-score (WAZ)	−0.65 ± 1.29	−0.64 ± 1.09
Length-for-age Z-score (LAZ)	−0.30 ± 1.70	−0.15 ± 1.46
Weight-for-length Z-score (WLZ)	−0.51 ± 0.95	−0.58 ± 1.05

**Table 2:** Anthropometry z-score changes between rice bran-fed and control infants. Data show changes in length-for-age (LAZ), weight-for-age (WAZ), and weight-for-length (WLZ) z-scores as well as hemoglobin (g/dL) at study timepoints 7 to 12 months compared to baseline (6 months). Controlling for sex and neighborhood. mos.= months of age. *P*-values were calculated by difference-in-differences analysis. Significant *p*-values (≤0.05) are bolded.

	6–7 Mos.	6–8 Mos.	6–9 Mos.	6–10 Mos.	6–11 Mos.	6–12 Mos.
**Length-for-Age z-Score (LAZ)**						
**Rice bran**	0.276	0.343	0.326	0.234	0.239	0.160
**Control**	0.049	0.098	0.137	0.028	−0.132	−0.284
**Difference**	0.227	0.245	0.189	0.205	0.371	0.444
***p*-value**	0.342	0.336	0.442	0.414	0.122	0.085
**Weight-for-age z-score (WAZ)**						
**Rice bran**	0.356	0.623	0.528	0.429	0.234	0.203
**Control**	0.125	0.207	0.095	−0.132	−0.383	−0.457
**Difference**	0.231	0.416	0.433	0.561	0.618	0.660
***p*-value**	0.190	**0.029**	**0.024**	**0.011**	**0.009**	**0.003**
**Weight-for-length z-score (WLZ)**						
**Rice bran**	0.218	0.535	0.392	0.286	0.011	−0.003
**Control**	0.052	0.145	−0.070	−0.323	−0.598	−0.670
**Difference**	0.166	0.390	0.462	0.609	0.609	0.667
***p*-value**	0.544	0.105	0.069	**0.023**	**0.013**	**0.008**
**Hemoglobin (g/dL)**						
**Rice bran**	0.201	0.218	0.396	0.405	−0.095	0.404
**Control**	−0.154	−0.254	−0.113	−0.454	−0.703	−0.355
**Difference**	0.355	0.472	0.508	0.859	0.608	0.759
***p*-value**	0.435	0.278	0.314	**0.031**	0.165	0.074

## Data Availability

Data available upon request from authors.

## Supplementary Materials

The following are available online at https://www.mdpi.com/article/10.3390/nu14030609/s1, Supplementary Figure S1: Z-score and hemoglobin changes; Supplementary Table S1: Total DBS collected monthly; Supplementary Table S2: List of DBS metabolites.

## References

[1] Chang S.M., Walker S.P., Grantham-McGregor S., Powell C.A. (2002). Early Childhood Stunting and Later Behaviour and School Achievement. J. Child Psychol. Psychiatry.

[2] Stein A.D., Wang M., Martorell R., Norris S.A., Adair L.S., Bas I., Sachdev H.S., Bhargava S.K., Fall C.H.D., Gigante D.P. (2010). Growth Patterns in Early Childhood and Final Attained Stature: Data from Five Birth Cohorts from Low- and Middle-Income Countries. Am. J. Hum. Biol..

[3] Sudfeld C.R., McCoy D.C., Danaei G., Fink G., Ezzati M., Andrews K.G., Fawzi W.W. (2015). Linear Growth and Child Development in Low- and Middle-Income Countries: A Meta-Analysis. Pediatrics.

[4] Victora C.G., Adair L., Fall C., Hallal P.C., Martorell R., Richter L., Sachdev H.S. (2008). Maternal and Child Undernutrition Study Group Maternal and Child Undernutrition: Consequences for Adult Health and Human Capital. Lancet.

[5] United Nation’s Children’s Fund (UNICEF) (2013). Improving Child Nutrition: The Achievable Imperative for Global Progress. https://data.unicef.org/resources/improving-child-nutrition-the-achievable-imperative-for-global-progress/.

[6] United Nations Children’s Fund (UNICEF) (2021). World Health Organization, International Bank for Reconstruction and Development/The World Bank. Levels and Trends in Child Malnutrition: Key Findings of the 2021 Edition of the Joint Child Malnutrition Estimates..

[7] Allen L.H. (2012). Global Dietary Patterns and Diets in Childhood: Implications for Health Outcomes. ANM.

[8] The World Health Organization (WHO) Global Strategy for Infant and Young Child Feeding. https://www.who.int/publications-detail-redirect/9241562218.

[9] The World Health Organization (WHO) Infant and Young Child Feeding. https://www.who.int/news-room/fact-sheets/detail/infant-and-young-child-feeding.

[10] Bai Y., Alemu R., Block S.A., Headey D., Masters W.A. (2021). Cost and Affordability of Nutritious Diets at Retail Prices: Evidence from 177 Countries. Food Policy.

[11] Dewey K.G., Adu-Afarwuah S. (2008). Systematic Review of the Efficacy and Effectiveness of Complementary Feeding Interventions in Developing Countries. Matern. Child Nutr..

[12] Gong Y.Y., Watson S., Routledge M.N. (2016). Aflatoxin Exposure and Associated Human Health Effects, a Review of Epidemiological Studies. Food Saf..

[13] Havelaar A.H., Kirk M.D., Torgerson P.R., Gibb H.J., Hald T., Lake R.J., Praet N., Bellinger D.C., de Silva N.R., Gargouri N. (2015). World Health Organization Global Estimates and Regional Comparisons of the Burden of Foodborne Disease in 2010. PLoS Med..

[14] United Nation’s Children’s Fund (UNICEF) (2021). The State of the World’s Children 2021. https://www.unicef.org/reports/state-worlds-children-2021.

[15] Adesogan A.T., Havelaar A.H., McKune S.L., Eilittä M., Dahl G.E. (2020). Animal Source Foods: Sustainability Problem or Malnutrition and Sustainability Solution? Perspective Matters. Glob. Food Secur..

[16] Hilborn R., Banobi J., Hall S.J., Pucylowski T., Walsworth T.E. (2018). The Environmental Cost of Animal Source Foods. Front. Ecol. Environ..

[17] Willett W., Rockström J., Loken B., Springmann M., Lang T., Vermeulen S., Garnett T., Tilman D., DeClerck F., Wood A. (2019). Food in the Anthropocene: The EAT–Lancet Commission on Healthy Diets from Sustainable Food Systems. Lancet.

[18] The Food and Agriculture Organization of the United Nations (FAO) (2017). Mali: Country Fact Sheet on Food and Agriculture Policy Trends. http://www.fao.org/3/a-i7617e.pdf.

[19] The Food and Agriculture Organization of the United Nations (FAO) (2018). Rice Market Monitor (RMM). http://www.fao.org/economic/est/publications/rice-publications/rice-market-monitor-rmm/en/.

[20] Borresen E.C., Ryan E.P., Watson R.R., Preedy V.R., Zibadi S. (2014). Chapter 22—Rice Bran: A Food Ingredient with Global Public Health Opportunities. Wheat and Rice in Disease Prevention and Health.

[21] Sharif M.K., Butt M.S., Anjum F.M., Khan S.H. (2014). Rice Bran: A Novel Functional Ingredient. Crit. Rev. Food Sci. Nutr..

[22] Zarei I., Brown D.G., Nealon N.J., Ryan E.P. (2017). Rice Bran Metabolome Contains Amino Acids, Vitamins & Cofactors, and Phytochemicals with Medicinal and Nutritional Properties. Rice.

[23] Zarei I., Luna E., Leach J.E., McClung A., Vilchez S., Koita O., Ryan E.P. (2018). Comparative Rice Bran Metabolomics across Diverse Cultivars and Functional Rice Gene–Bran Metabolite Relationships. Metabolites.

[24] Gul K., Yousuf B., Singh A.K., Singh P., Wani A.A. (2015). Rice Bran: Nutritional Values and Its Emerging Potential for Development of Functional Food—A Review. Bioact. Carbohydr. Diet. Fibre.

[25] Dipti S.S., Bergman C., Indrasari S.D., Herath T., Hall R., Lee H., Habibi F., Bassinello P.Z., Graterol E., Ferraz J.P. (2012). The Potential of Rice to Offer Solutions for Malnutrition and Chronic Diseases. Rice.

[26] Kurtys E., Eisel U.L.M., Hageman R.J.J., Verkuyl J.M., Broersen L.M., Dierckx R.A.J.O., de Vries E.F.J. (2018). Anti-Inflammatory Effects of Rice Bran Components. Nutr. Rev..

[27] Saji N., Francis N., Schwarz L.J., Blanchard C.L., Santhakumar A.B. (2020). The Antioxidant and Anti-Inflammatory Properties of Rice Bran Phenolic Extracts. Foods.

[28] Henderson A.J., Kumar A., Barnett B., Dow S.W., Ryan E.P. (2012). Consumption of Rice Bran Increases Mucosal Immunoglobulin A Concentrations and Numbers of Intestinal Lactobacillus Spp. J. Med. Food.

[29] Kumar A., Henderson A., Forster G.M., Goodyear A.W., Weir T.L., Leach J.E., Dow S.W., Ryan E.P. (2012). Dietary Rice Bran Promotes Resistance to Salmonella Enterica Serovar Typhimurium Colonization in Mice. BMC Microbiol..

[30] Yang X., Wen K., Tin C., Li G., Wang H., Kocher J., Pelzer K., Ryan E., Yuan L. (2014). Dietary Rice Bran Protects against Rotavirus Diarrhea and Promotes Th1-Type Immune Responses to Human Rotavirus Vaccine in Gnotobiotic Pigs. Clin. Vaccine Immunol..

[31] Yang X., Twitchell E., Li G., Wen K., Weiss M., Kocher J., Lei S., Ramesh A., Ryan E.P., Yuan L. (2015). High Protective Efficacy of Rice Bran against Human Rotavirus Diarrhea via Enhancing Probiotic Growth, Gut Barrier Function and Innate Immunity. Sci. Rep..

[32] Lim M.D. (2018). Dried Blood Spots for Global Health Diagnostics and Surveillance: Opportunities and Challenges. Am. J. Trop. Med. Hyg..

[33] Zambrana L.E., McKeen S., Ibrahim H., Zarei I., Borresen E.C., Doumbia L., Boré A., Cissoko A., Douyon S., Koné K. (2019). Rice Bran Supplementation Modulates Growth, Microbiota and Metabolome in Weaning Infants: A Clinical Trial in Nicaragua and Mali. Sci. Rep..

[34] Ryan E., Baxter B., Li K., Wolfe L., Yao L., Broecling C., Borreson E., Zhang L., Zarei I., Beale M. (2020). Developing Biomarkers of Rice Bran and Navy Bean Intake via Integrated Metabolomics from Infants, Children and Adults for Association with Gut Health Properties. Curr. Dev. Nutr..

[35] Hesham M.S. (2004). Intestinal Parasitic Infections and Micronutrient Deficiency: A Review. Med. J. Malays..

[36] Ford L., Kennedy A.D., Goodman K.D., Pappan K.L., Evans A.M., Miller L.A.D., Wulff J.E., Wiggs B.R., Lennon J.J., Elsea S. (2020). Precision of a Clinical Metabolomics Profiling Platform for Use in the Identification of Inborn Errors of Metabolism. J. Appl. Lab. Med..

[37] Storey J.D., Tibshirani R. (2003). Statistical Significance for Genomewide Studies. Proc. Natl. Aacd. Sci. USA.

[38] Martinez-Moral M.-P., Kannan K. (2019). Allantoin as a Marker of Oxidative Stress: Inter- and Intraindividual Variability in Urinary Concentrations in Healthy Individuals. Environ. Sci. Technol. Lett..

[39] Dingledine R., McBain C.J. (1999). Glutamate and Aspartate Are the Major Excitatory Transmitters in the Brain. Basic Neurochemistry: Molecular, Cellular and Medical Aspects.

[40] Gonzalez-Riano C., Garcia A., Barbas C. (2016). Metabolomics Studies in Brain Tissue: A Review. J. Pharm. Biomed. Anal..

[41] Moreau G.B., Ramakrishnan G., Cook H.L., Fox T.E., Nayak U., Ma J.Z., Colgate E.R., Kirkpatrick B.D., Haque R., Petri W.A. (2019). Childhood Growth and Neurocognition Are Associated with Distinct Sets of Metabolites. eBioMedicine.

[42] Zhou Y., Danbolt N.C. (2014). Glutamate as a Neurotransmitter in the Healthy Brain. J. Neural Transm..

[43] Paredes S.D., Barriga C., Reiter R.J., Rodríguez A.B. (2009). Assessment of the Potential Role of Tryptophan as the Precursor of Serotonin and Melatonin for the Aged Sleep-Wake Cycle and Immune Function: Streptopelia Risoria as a Model. Int. J. Tryptophan Res..

[44] Sanders L.M., Zeisel S.H. (2007). Choline: Dietary Requirements and Role in Brain Development. Nutr. Today.

[45] Zeisel S.H., da Costa K.-A. (2009). Choline: An Essential Nutrient for Public Health. Nutr. Rev..

[46] Thompson R.A., Nelson C.A. (2001). Developmental Science and the Media. Early Brain Development. Am. Psychol..

[47] Gao J., Xu K., Liu H., Liu G., Bai M., Peng C., Li T., Yin Y. (2018). Impact of the Gut Microbiota on Intestinal Immunity Mediated by Tryptophan Metabolism. Front. Cell. Infect. Microbiol..

[48] Perna S., Alalwan T.A., Alaali Z., Alnashaba T., Gasparri C., Infantino V., Hammad L., Riva A., Petrangolini G., Allegrini P. (2019). The Role of Glutamine in the Complex Interaction between Gut Microbiota and Health: A Narrative Review. Int. J. Mol. Sci..

[49] Dai Z.-L., Wu G., Zhu W.-Y. (2011). Amino Acid Metabolism in Intestinal Bacteria: Links between Gut Ecology and Host Health. Front. Biosci..

[50] Pollitt E. (2000). A Developmental View of the Undernourished Child: Background and Purpose of the Study in Pangalengan, Indonesia. Eur. J. Clin. Nutr..

[51] Liguori I., Russo G., Curcio F., Bulli G., Aran L., Della-Morte D., Gargiulo G., Testa G., Cacciatore F., Bonaduce D. (2018). Oxidative Stress, Aging, and Diseases. Clin. Interv. Aging.

[52] Pizzino G., Irrera N., Cucinotta M., Pallio G., Mannino F., Arcoraci V., Squadrito F., Altavilla D., Bitto A. (2017). Oxidative Stress: Harms and Benefits for Human Health. Oxidative Med. Cell. Longev..

[53] Iannotti L.L., Zavaleta N., León Z., Shankar A.H., Caulfield L.E. (2008). Maternal Zinc Supplementation and Growth in Peruvian Infants. Am. J. Clin. Nutr..

[54] Rahman M.M., Tofail F., Wahed M.A., Fuchs G.J., Baqui A.H., Alvarez J.O. (2002). Short-Term Supplementation with Zinc and Vitamin A Has No Significant Effect on the Growth of Undernourished Bangladeshi Children. Am. J. Clin. Nutr..

[55] Stewart C.P., Caswell B., Iannotti L., Lutter C., Arnold C.D., Chipatala R., Prado E.L., Maleta K. (2019). The Effect of Eggs on Early Child Growth in Rural Malawi: The Mazira Project Randomized Controlled Trial. Am. J. Clin. Nutr..

[56] Zambrana L.E., Weber A.M., Borresen E.C., Zarei I., Perez J., Perez C., Rodríguez I., Becker-Dreps S., Yuan L., Vilchez S. (2021). Daily Rice Bran Consumption for 6 Months Influences Serum Glucagon-Like Peptide 2 and Metabolite Profiles without Differences in Trace Elements and Heavy Metals in Weaning Nicaraguan Infants at 12 Months of Age. Curr. Dev. Nutr..

[57] Li K., Naviaux J.C., Monk J.M., Wang L., Naviaux R.K. (2020). Improved Dried Blood Spot-Based Metabolomics: A Targeted, Broad-Spectrum, Single-Injection Method. Metabolites.

[58] Moreno-Torres M., García-Llorens G., Moro E., Méndez R., Quintás G., Castell J.V. (2021). Factors That Influence the Quality of Metabolomics Data in in Vitro Cell Toxicity Studies: A Systematic Survey. Sci. Rep..

