# Supplementary material for: Non-Targeted Dried Blood Spot-Based Metabolomics Analysis Showed Rice Bran Supplementation Effects Multiple Metabolic Pathways during Infant Weaning and Growth in Mali
Source: Nutrients. 2022 Jan 30;14(3):609. doi: 10.3390/nu14030609 (PMC8840250; doi:10.3390/nu14030609)
Supplement: Supplementary file 1 [file nutrients-14-00609-s001.zip › Figure S1. Z-score and hemoglobin changes.pdf]

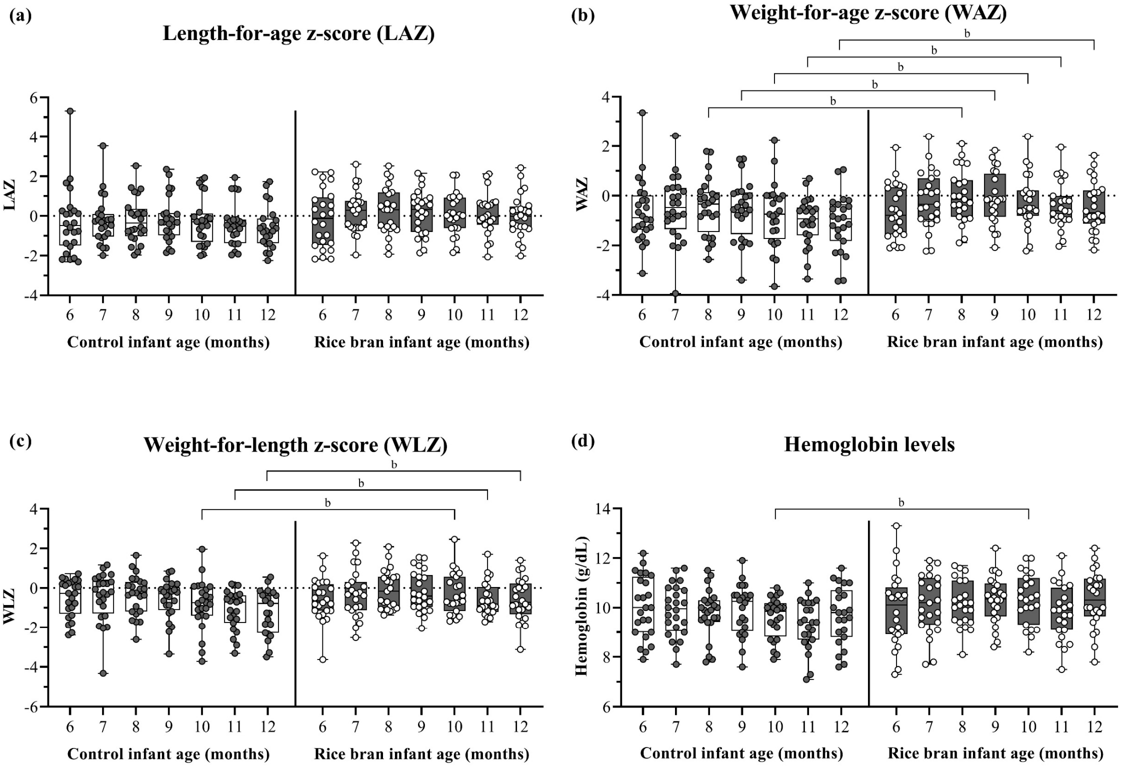

Figure S1. Anthropometric z-score and hemoglobin changes in Malian weaning infants from baseline in rice bran-fed relative to control infants. Controlling for sex and neighborhood. (**a**) No significant length-for-age z-score (LAZ) changes. (**b**) Significant weight-for-age z-score (WAZ) changes at 8, 9, 10, 11, and 12 months of age. (**c**) Significant length-for-age z-score (WLZ) changes at 10, 11, and 12 months of age. (**d**) Significant change in hemoglobin (g/dL) at 10 months of age. b=significant difference in fold change ($p \leq 0.05$) between control and rice bran groups. P-values were calculated by difference-in-differences analysis.
